# Evaluation of skin diseases and disorders in photographers

**DOI:** 10.4103/0019-5278.55126

**Published:** 2009-08

**Authors:** M. S. Attarchi, S. Mohammadi, E. Asghari

**Affiliations:** Assistant Professor of Occupational Medicine, Department of Occupational Medicine and Occupational medicine research center of Iran University of Medical Sciences, Tehran, Iran; Occupational Medicine Specialist, University of Amsterdam- Academic Medical Center-Coronel Institute of Occupational Health, Amsterdam, Netherlands

**Keywords:** Occupational exposure, skin disease, contact dermatitis, hydroquinone

## Abstract

Occupational skin disease is very common and important among photographers due to the chemical substances used in photographic processes. In this cross-sectional study, 100 photographers were asked about their occupational exposures in their workplace. Physical examinations were done to find skin diseases and information about demographic factors and history of other skin diseases was collected via a questionnaire. This study examined 100 people, 86 men and 14 women; 37% of these 100 subjects were found to suffer from skin diseases and disorders: 24% contact dermatitis, 2% leukoderma, 3% nail hyperpigmentation. Less than half of these subjects (43%) were found to be working with nonmechanized (manual) printers whereas the other 57% worked with computerized printers. Employees working with nonmechanized printers were found to have a statistically meaningful increase in skin diseases compared with subjects who were working with computerized printers (Odds ratio = 7.4, 95% CI = 2.59−21.92, *P* = 0.001). Some (41%) of these subjects did not use gloves and were found to have a statistically significant increased incidence of skin diseases compared with the ones who used gloves (Odds ratio = 4.11, 95% CI = 1.72–13.21, *P* value = 0.002). Generally, it seems that adequate ventilation and protective gloves are necessary for decreasing the prevalence of occupational skin diseases among photographers. Also, educating the photographers about the risks of the chemical substances in their workplace is very important.

## INTRODUCTION

Skin disease arising from occupational exposure is common and second only to musculoskeletal disorders as a cause of occupational ill health.[1–5] Contact dermatitis is the most common occupational skin disease[6–8] and still one of the most common causes of job transfer in workers.[9,10] Also, occupational leukodermas have special importance in occupational skin diseases.[11]

The first report of occupational leukodermas which were caused by phenol, catechol, and hydroquinone was published by Oliver and Schwartz in 1939.[12] Later, several studies about the effects of industrial chemical agents on skin pigmentation had been performed.[13–20] These agents cause depigmentation and have selective toxicity against melanocytes.[21–25] Photography is a basic industry and has an important role in lithography, printing, medicine, electronic industry, police activities, etc.[26] Photographers are in direct contact with different hazardous chemicals during the developing and fixation process of the photographs. The most common routes of exposure are skin contact and inhalation.[26,27]

Chemical agents used in the developing process are acids and alkalis, solvents, hydroquinone, *p*-phenylendiamine, acetic acid, hydrochloric acid, chromate, selenium, and formaldehyde.[28,29]

Following exposure to these chemicals, photographers become susceptible to a variety of skin diseases and disorders such as burns (due to acids, bases, chromates), contact dermatitis (due to acids, alkalis, *p*-phenylendiamine), lichen-planus and lichen-planus-like eruptions (due to *p*-phenylendiamine) and leukodermas (due to hydroquinone).[26,30]

## MATERIALS AND METHODS

This study was done as a cross-sectional study in 2007 in Tehran by selecting 100 phototgraphy shops by a simple, randomized sampling method. One employee was chosen from the workers involved in photo printing, based on the alphabetical order of the first letter of all the family names. At first, these persons were asked to complete a questionnaire containing demographic and occupational data such as age, gender, duration of working as a photographer, type of printing method (manual or computerized), existing occupational skin diseases and treatments, history of any kinds of skin diseases, history of allergies and atopy, using gloves, and personal habits. They were then examined physically.

In this study the definition of skin disease were the presence of signs of acute contact dermatitis (rash, erythema, vesicle, skin ulcer) or chronic contact dermatitis (skin thickening, fissure), leukodermas, hyperpigmentation, lichen-planus, and onycholysis besides the history of recovery subsequent to the discontinuation of occupational exposure.

The base of diagnosis was the physical examination of the study participants by two occupational medicine specialists in collaboration with one of the dermatology professors. This study was approved by the Ethics Committee of the Iran University of Medical Sciences. All of the data were processed by SPSS-16 statistical software.

## RESULTS

In this study, 86 participants were male and 14 were female. The mean age was 37.1 years with a range of 18 to 72 years. The mean number of years spent in developing photos was 15.5 years with a range of 0.5 to 56 years.

In this study, 33 persons had different skin diseases, the most common one being contact dermatitis (24 persons). Data pertaining to the prevalence of skin diseases according to the type of printing method are shown in Table 1.

**Table 1:** Prevalence of skin diseases according to type of printing method

Skin disease	Frequency (Prevalence)		
	
	Manual	Computerized	Total
Contact dermatitis	17(17)	7(7)	24(24)
Onycholysis	2(2)	0(0)	2(2)
Leukoderma	2(2)	0(0)	2(2)
Nail hyperpigmentation	3(3)	0(0)	3(3)
Mouth aphthous	2(2)	0(0)	2(2)
Generalized pruritus	0(0)	1(1)	1(1)
Seborrheic dermatitis	0(0)	1(1)	1(1)
Alopecia areata	1(1)	1(1)	2(2)
Total	27	10	37

Figures are in parentheses are in percentage

There were two cases of leukoderma, one of them was a 38 year-old man with a history of working with nonmechanized (manual) developing processes of photos for 15 years and had two hypopigmented lesions on the dorsal part of his hand 1.5 cm × 1.5 cm. The lesions had developed six years after beginning his job and he had a history of chronic contact dermatitis before the development of these hypopigmented lesions. He had begun using latex gloves two years ago after which the healing of the lesions accelerated.

The second case was a 28 year-old man with a nine-year history of working in manual developing and printing of photos and had hypopigmented lesions on the dorsum of his right hand and fingers; the lesions had developed seven years after beginning his job.

By using the Fisher exact test, it was concluded that skin disease had no relationship with sex (*P* = 0.134).

In 69% of all cases, involvement was in the hands and the fingers (distal to the wrist).

The mean time of onset of skin disease was 2.1 years after beginning the work with a range of one month to 14 years; the standard deviation (SD) was 3.3 months. In this study, the onset of skin diseases in the first year of work was 66.7%.

Forty-one study participants did not use gloves so that their hands were in direct contact with photo developing agents, 29 persons used latex gloves, 26 persons used plastic gloves, and four persons used clips. Less than half (41%) of the participants did not use gloves and they were found to have a statistically significant increased incidence of skin diseases compared with the ones who used gloves (Odds ratio = 4.11, 95% CI = 1.72–13.21, *P* = 0.002).

By using the chi-square test, it was shown that persons who did not use gloves had significantly more risk for skin diseases than the group using latex gloves (Odds ratio = 14.81, 95% CI = 2.7–99, *P* = 0.0001), but there was no significant difference between the group using plastic gloves and not using gloves (Odds ratio = 2.67, 95% CI = 0.44–2.96, *P* = 0.23).

Forty-three subjects used manual printing and 57 used computerized printers; the chi-square test showed that the manual printing group had significantly more skin diseases than the computerized printing group (Odds ratio = 7.4, 95% CI = 2.59–21.92 and *P* = 0.001). Among all the participants, two persons had seasonal allergies and two developed urticaria after eating almonds. Fourteen subjects had dyspnea, one of them had hoarseness, and the other had epistaxis during the process of developing the photos.

## DISCUSSION

Photographers are susceptible to different skin diseases like contact dermatitis (due to acids, alkalis), lichen-planus and lichen-planus-form eruptions (due to *p*-phenylendiamine) and leukoderma (due to hydroquinone) due to their contact with different agents.[1,2,27] These diseases were reported in previous studies also.[28,30–33]

Overall, 33% of photographers in our study had skin diseases and at least 30% of them were occupation-related (one case was of seborrheic dermatitis and two cases were of alopecia areata). In other studies, this rate was reported to be 42–45% but the rates according to our study findings were still lower than previous reports.[27,30] On the other hand, the most common disease among film developers was contact dermatitis, which was consistent with findings from previous studies.[28,31]

Our study found that 24% of the photographers had occupational contact dermatitis; other studies had reported a 25–38% rate of involvement.[27,30,31]

The lower percentage of women (2 vs 31% men) affected by skin diseases is due to their lower number in this study.

It is notable that in most photographers with contact dermatitis, their disease disappeared partially or completely if the exposure to chemical agents was discontinued.

Contact dermatitis can not only cause problems by itself, but it can also make the patient prone to other skin diseases like leukodermas.[34–36]

As mentioned in this study, the onset of most of the skin diseases (66.7%) was in the first year of work. In previous studies, the rate of involvement in the first year was reported to be 45%.[1] The reason for this early onset could be inexperience and unawareness in the workers. Education about the risks of chemical substances and their effects on the skin, and training the photographers in the beginning of the job would have important roles in decreasing the contact of the employees with these agents.

Contact with catechols and hydroquinone can result in leukodermas in photographers. Phenol, hydroquinone, and catechols resemble tyrosine and the selective melanocytotoxicity results in inhibition of melanin production. Also, tyrosinase oxidizes these agents (phenol, hydroquinone, and catechols) and leads to the formation of free radicals which initiate peroxidation. Peroxidation can destroy melanocyte lipoprotein membranes and induce abnormal melanogenesis.[2]

Differentiation of occupational and idiopathic leukodermas, especially based on just clinical manifestations, is difficult.

Idiopathic leukoderma is seen usually in the second decade of life and in 30% of the cases, the patient has a positive family history and the lesions are usually located symmetrically in the dorsal parts of the hands and forearms, and around the mouth and the eyes. Usually, there is a concomitant occurrence of autoimmune disease.[27]

Occupational leukoderma is limited to a part of the body which is exposed to the chemical agent (usually the hands and the elbows).[2,37]

As noted, leukoderma can be seen following the contact dermatitis, which emphasizes the importance of protection from contact dermatitis.

In this study, several years after contact dermatitis, leukodermas were seen in the same place in one case. Also, in both leukoderma patients, lesions were limited to the dorsum of the hand and were asymmetric; the patients did not have any familial history or any history of autoimmune disease.

Three cases of nail hyperpigmentation were seen in which the color of the nails became normal by discontinuing the exposure in the beginning of the course of hyperpigmentation, but after a period of time, the nail hyperpigmentation (to brownish yellow) became permanent. This finding was in agreement with those of previous studies.[1,38]

Two cases of onycholysis were seen in which all fingers of both hands were involved. In these cases, fungal and infectious diseases were ruled out by paraclinical studies and the onset of the lesion was simultaneous with using the chemical agents during the developing process.

Skin diseases were more common in photographers who used manual printing than in the ones with computerized printers. This emphasizes the necessity of automation of the photo developing process to decrease the incidence of occupational skin diseases. Of course, different substances used in computerized printing can also cause different skin diseases (the agents could enter the system during transportation and be systemically absorbed).

Regardless of whether the subjects were involved with color or black and white printing, unless some of the diseases (like lichen-planus), because of the same agents being used and the cross-reaction between these agents, skin diseases were seen in both groups.[38,39]

In the case of hand protection during contact with the chemical agents, employees who used latex gloves had less contact dermatitis than the ones who did not use gloves. Thus, gloves are necessary in developing photos and it is better to use antiacid nitryl gloves during the transport of acid agents, and neoprene gloves in the rest of the process. Gloves should be checked daily for tears.

Overall, paying attention to factors like environmental hygiene, adequate ventilation, and using suitable gloves during the developing process and transportation of chemical agents are obligatory to decrease the rate of occupational skin diseases. Also, automation of the photo developing process is an important factor in decreasing the incidence of skin diseases in photographers. Training and education of the photographers about the risks of chemical substances and the importance of decreasing their contact with these agents have an important role in decreasing the rate of occupational skin diseases.[40]
